# Rupture of uterine artery pseudoaneurysm: role of ultrasonography in postpartum hemorrhage management

**DOI:** 10.11604/pamj.2016.25.136.10676

**Published:** 2016-11-04

**Authors:** Narjes Karmous, Amira Ayachi, Sadok Derouich, Lassaad Mkaouar, Mechaal Mourali

**Affiliations:** 1 Department of Obstetrics and Gynecology, University Hospital of Bougatfa, Bizerte, Tunisia; 2 Faculty of Medicine of Tunis, University Tunis El Manar, Tunisia

**Keywords:** Postpartum hemorrhage, cesarean delivery, uterine artery pseudoaneurysm, transcatheter arterial embolization

## Abstract

Uterine artery pseudoaneurysm (UAP) rupture should be considered in case of late genital bleeding without obvious cause and lead to perform a sonographic examination with Doppler-scan. We report two cases of late post-partum hemorrhage from UAP diagnosed as such using color Doppler US. In order to avert life-threatening bleeding, prompt and accurate diagnosis should be made using color Doppler US since the latter plays a significant role in demonstrating the vascular nature of this anechoic uterine lesion.

## Introduction

A pseudoaneurysm is defined as dilation of an artery with partial or full disruption of the vessel wall [1, 2]. Without precise ultrasonographic and radiologic diagnosis before the manifestation of symptoms associated with hemorrhage [3], these pseudoaneurysms are prone to unpredictable rupture, resulting in exsanguination with high morbidity and mortality rates [4]. The 24th edition of Williams Obstetrics described UAP rupture as a possible cause of secondary postpartum hemorrhage. A recent large study showed that secondary PPH is associated with 2.3/1000 deliveries [5] consistent with data that UAP rupture occurs in 2.3/1000 deliveries [6]. This diagnosis should be considered in case of late genital bleeding without obvious cause and lead to perform a sonographic examination with Doppler-scan before diagnostic and therapeutic arteriography [7]. Color Doppler US plays a significant role in demonstrating the vascular nature of these anechoic uterine lesions. We describe two cases of late post-partum hemorrhage from UAP diagnosed as such using color Doppler US, along with a review of the literature reporting UAP. Our purpose is to assess the efficiency of color Doppler US in the management of severe postpartum hemorrhage induced by a ruptured pseudoaneurysm.

## Patient and observation

**Figure 1:**
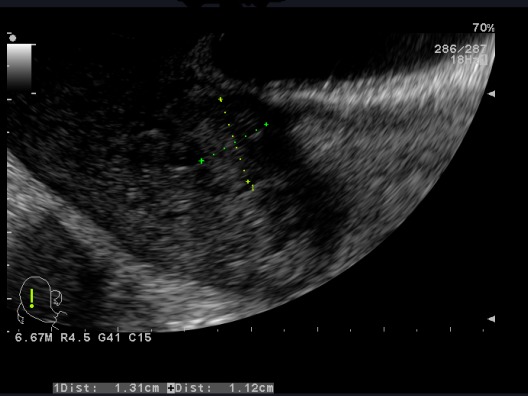
Uterine artery pseudoaneurysm; Transvaginal US shows anechoic masses at the uterine cervix

**Figure 2:**
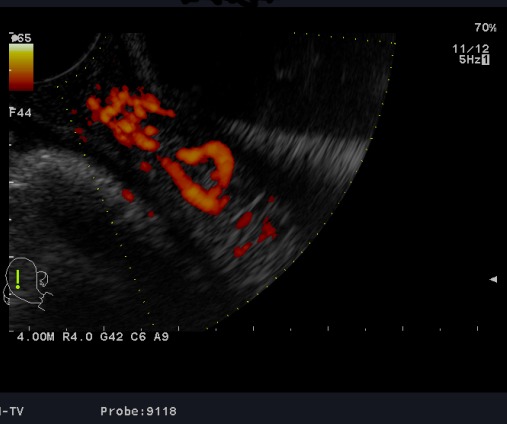
Uterine artery pseudoaneurysm; examination with color Doppler transvaginal US shows spining blood flow

**Figure 3:**
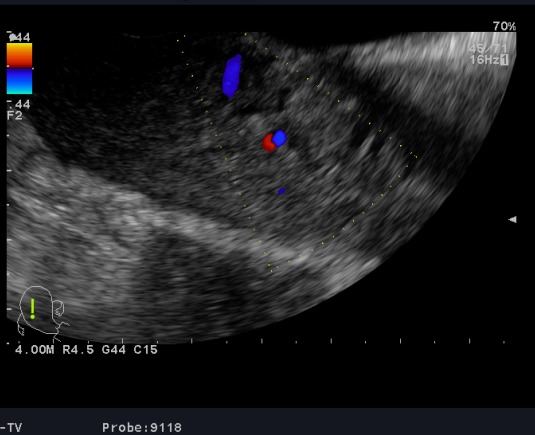
Color Doppler US

**Figure 4:**
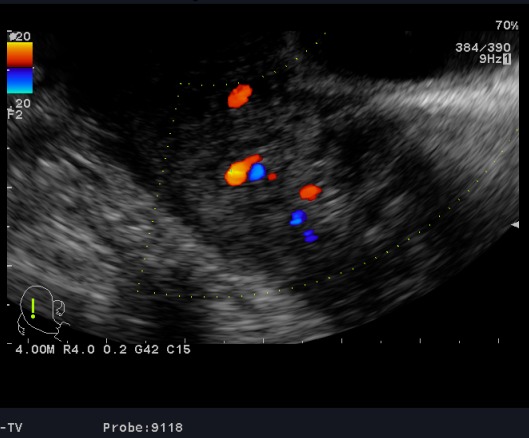
“Yin-yang” mosaic pattern

**Observation 1:** A 24-years-old woman (gravid 1 para 1) with no previous disease history underwent an emergency cesarean section (CS) at 38 weeks of gestation because of failed vaginal delivery. The clinical course after CS was uncomplicated, and the patient was discharged to home. She came to our emergency 32 days after CS because of abnormal uterine bleeding. On arrival at our emergency, massive uterine bleeding was observed and vital signs were unstable with blood pressure of 9/6 cm Hg and heart rate of 105 bpm. The hemoglobin concentration was 5.3g/dL. Crystalloid solution was administered, and 3 units of concentrated red blood cells were transfused. Transvaginal US revealed an anechoic lesion at the uterine cervix measuring 13×11 mm in diameter (Figure 1). Color Doppler transvaginale US showed a spinning blood flow in the previously indicated anechoic structure (Figure 2, Figure 3). It also showed a “yin-yang” mosaic pattern indicating the presence of a pseudoaneurysm (Figure 4). However, no retained gestational products were detected. Patient was treated with bilateral internal iliac artery ligation because emergency transcatheter embolization was not available in our facility. The hemorrhage ceased and the course was uneventful. The patient was discharged 2 days later. The patient returned to our emergency 49 days after CS because of continuous abnormal uterine bleeding. On examination, there was profuse bleeding; vital signs remained stable with blood pressure of 12/7 cm Hg and heart rate of 87bpm. The hemoglobin concentration was 8 g/dL. An attempt was made to neutralize the uterine bleeding using the balloon of a urinary catheter. The bleeding decreased but was still present. It was decided to proceed with uterine artery embolization (UAE). After informed consent was obtained, digital subtraction angiography was performed. On pelvic angiogram, the major feeding artery to pseudoaneurysm was confirmed to be the left uterine artery. After embolization, a complete angiogram was obtained to confirm that occlusion of the bilateral uterine artery has been performed to induce thrombosis of the pseudoaneurysm. UAE was successful, and complete hemostasis was achieved. The patient was discharged 4 days after embolization. She has had normal menstruation periods and no abnormal uterine bleeding since. No further lesion has been detected at transvaginal US.

**Figure 5:**
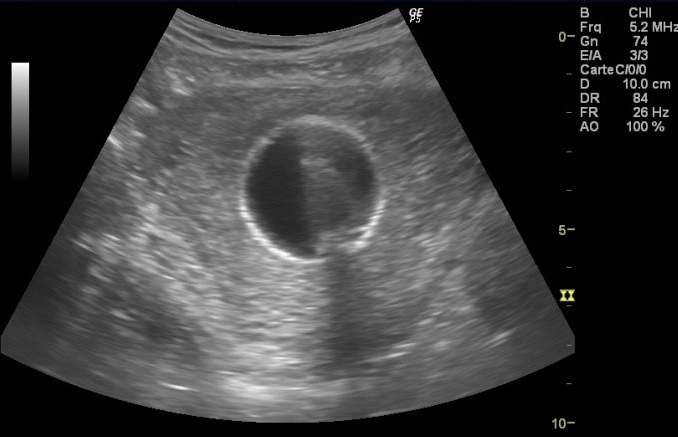
Transvaginal grey-scale US showed the urinary catheter balloon in place

**Figure 6:**
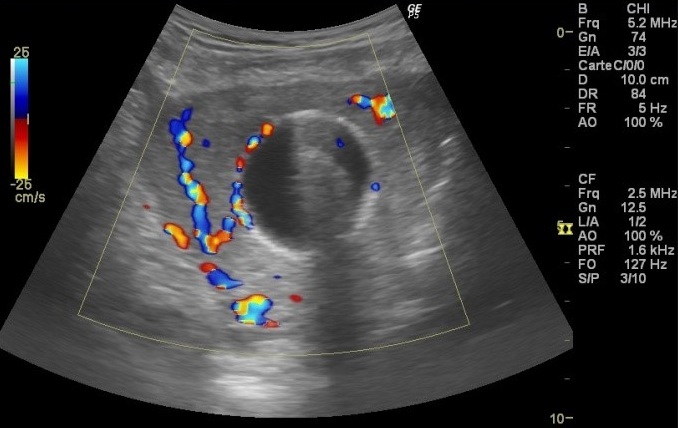
Color Doppler transvaginal US revealed the presence of a right uterine artery pseudoaneurysm

**Figure 7:**
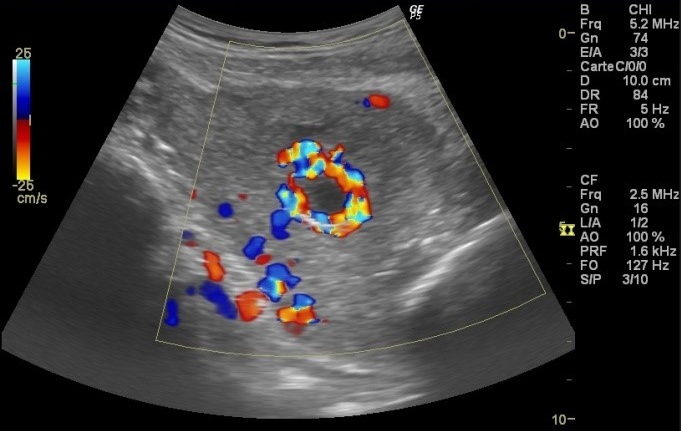
Spinning blood flow around the urinary catheter balloon

**Observation 2:** A 33-years-old woman presented to our emergency department with vaginal bleeding. 20 days earlier, after a pregnancy without complications, she underwent a delivery by cesarean section because of failure to progress. The clinical course after CS was without further complications. Physical examination revealed profuse amount of active bleeding. Vital signs were unstable with blood pressure of 8/7 cm Hg and heart rate of 110 bpm. The hemoglobin concentration was 6.3 g/dL. Crystalloid solution was administered and 2 units of concentrated red blood cells were transfused. An attempt was made to neutralize the uterine bleeding using the balloon of a urinary catheter and it was successful. Transvaginal grey-scale US showed the urinary catheter balloon in place (Figure 5). Color Doppler transvaginal US revealed the presence of a right UAP by demonstrating a spinning blood flow around the urinary catheter balloon (Figure 6, Figure 7). At the time of writing this report, UAE has been planned.

## Discussion

UAP has long been considered as a rare disorder, causing massive PPH similar to our two present cases. It occurs more frequently than considered: indeed, it occurs at a rate of 0.2% [6]. Many clinical interventions have been postulated to be responsible for development of UAP. The most frequent cause of UAP was CS, accounting for 25 of 57 cases (47.4%) [8]. This would be reasonable because CS is prone to cause vessel injuries involving branches of the uterine arteries [8]. The clinical appearance of a pseudoaneurysm is variable, precise diagnosis of pseudoaneurysm in an asymptomatic patient is difficult [2]. Hemorrhagic shock can occur and may be the circumstance of discovering, but first diagnosis is often wrong [9]. Regarding diagnostic imaging modalities for pseudoaneurysm, the initial usefulness of US is well documented [2]. In general, on grayscale US, pseudoaneurysm has a characteristic sonographic appearance consisting of a pulsating anechoic or hypoechoic well defined cystic structure with or without associated pelvic hematoma or free fluid [10]. Within the pseudoaneurysm, swirling arterial flow with different directions and velocities is observed, with varying colors according to the variable degree of turbulence at color Doppler US. In the neck of the pseudoaneurysm, the “yin-yang” pattern may be potentially identified at duplex Doppler US because the arterial blood flows like a jet (forward flow) into the aneurysm cavity during systole, then reverses (backward flow) into the original artery during diastole [11].

This pattern is explained by the pressure gradient between a distended, high-pressure pseudoaneurysm and a low-pressure artery during diastole [1]. However, in the case of a UAP, visualization of the neck of the pseudoaneurysm at US may be difficult because of the small size of the parent artery [12]. Some authors had completed ultrasononography by angio computed tomodensitometry to confirm the diagnosis and to perform embolization in the same time [13, 14]. In the present cases, color Doppler US helped to establish the diagnosis of UAP by displaying turbulent blood flow with the “yin-yang” mosaic pattern after initial identification on grayscale US. In an analysis of 17 UAP cases, it was revealed that the uterine anechoic areas strongly suggest the presence of UAP. Subsequent definite diagnosis was made at angiography in 7 cases (41.2%), CT in 5 (29.4%), or color Doppler US in 5 (29.4%) [15]. This review indicates the importance of considering UAP in the differential diagnosis of postpartum hemorrhage and abnormal uterine bleeding after gynecologic or obstetric procedures. The unexpected appearance of uterine anechoic lesions at transvaginal US may suggest the presence of UAP. Prompt and accurate diagnosis should be made using color Doppler US to avert life-threatening bleeding since it plays a significant role in demonstrating the vascular nature of this anechoic uterine lesion.

## Conclusion

The present cases of UAP illustrate a rare vascular complication after CS. It is important to recognize this uterine vascular abnormality as a surgical complication. Ultrasonography is the most commonly performed initial imaging examination for evaluation of abnormal uterine bleeding. Color and duplex Doppler US are useful in the early diagnosis of UAP which is potentially a lethal disorder.
